# Investigating Properties of the Cardiovascular System Using Innovative Analysis Algorithms Based on Ensemble Empirical Mode Decomposition

**DOI:** 10.1155/2012/943431

**Published:** 2012-08-02

**Authors:** Jia-Rong Yeh, Tzu-Yu Lin, Yun Chen, Wei-Zen Sun, Maysam F. Abbod, Jiann-Shing Shieh

**Affiliations:** ^1^Research Center for Adaptive Data Analysis & Center for Dynamical Biomarkers and Translational Medicine, National Central University, Jhongli 3200, Taiwan; ^2^Department of Mechanical Engineering, Yuan Ze University, 135 Yuan-Tung Road, Chung-Li, Taoyuan 320, Taiwan; ^3^Department of Anesthesiology, Far Eastern Memorial Hospital, Taipei 220, Taiwan; ^4^Department of Surgery, Far Eastern Memorial Hospital, Taipei 22060, Taiwan; ^5^Department of Chemical Engineering & Materials Science, Yuan Ze University, Taoyuan 320, Taiwan; ^6^Department of Anesthesiology, College of Medicine, National Taiwan University, Taipei 100, Taiwan; ^7^School of Engineering and Design, Brunel University, London UB83PH, UK

## Abstract

Cardiovascular system is known to be nonlinear and nonstationary. Traditional linear assessments algorithms of arterial stiffness and systemic resistance of cardiac system accompany the problem of nonstationary or inconvenience in practical applications. In this pilot study, two new assessment methods were developed: the first is ensemble empirical mode decomposition based reflection index (EEMD-RI) while the second is based on the phase shift between ECG and BP on cardiac oscillation. Both methods utilise the EEMD algorithm which is suitable for nonlinear and nonstationary systems. These methods were used to investigate the properties of arterial stiffness and systemic resistance for a pig's cardiovascular system via ECG and blood pressure (BP). This experiment simulated a sequence of continuous changes of blood pressure arising from steady condition to high blood pressure by clamping the artery and an inverse by relaxing the artery. As a hypothesis, the arterial stiffness and systemic resistance should vary with the blood pressure due to clamping and relaxing the artery. The results show statistically significant correlations between BP, EEMD-based RI, and the phase shift between ECG and BP on cardiac oscillation. The two assessments results demonstrate the merits of the EEMD for signal analysis.

## 1. Introduction

Arterial stiffness is a powerful physiological marker of cardiovascular morbidity and mortality. However, the cardiovascular system is a complicated system which has effects of multiple underlying mechanisms. Correlations among systolic arterial pressure (SAP), arterial stiffness, and systemic resistance are significant topics for cardiovascular system. Moreover, since a cardiovascular system is nonlinear and nonstationary, the characteristics of the system should be assessed by suitable algorithms based on innovative signal processing techniques for such a nonlinear system. Therefore, two methods were developed to assess the arterial stiffness and systemic resistance of a cardiovascular system based on ensemble empirical mode decomposition (EEMD) technique. EEMD is an innovative signal processing algorithm developed to decompose intrinsic mode functions from a nonlinear and nonstationary time series [1].

In this study, for the purpose of obtaining a sequence of changes in the blood pressure, such as increasing then steady high blood pressure for SAP, arterial stiffness, and systemic resistance in a cardiovascular system, an experimental surgical operation has been conducted on a healthy young pig. In such an experiment, the clamping of intestine artery stimulated an acute rising of SAP and the relaxing of arterial clamping reversed the reaction to arterial clamping. Changes in SAP stimulated corresponding changes on arterial stiffness and systemic resistance of the cardiovascular system [2, 3]. This procedure has provided the material for the investigation so that a better understanding of the connections between SAP, arterial stiffness, and systemic resistance of the cardiovascular system can be realized.

Previous studies have shown that augmentation index (AIx) and reflection index (RI) provide as good indicators for aortic stiffness [4–6], which can be calculated as the ratios between the amplitudes of forward wave, reflected wave and systolic peak. AxI is determined by both the magnitude and timing of the reflected wave [6]. Furthermore, a more accurate measurement can be obtained after separating the BP signal into its forward and reflected components, which requires an extra measurement of aortic flow. Previously, Westerhof et al. presented a new method to quantify the magnitude of reflection independent of the time of the reflected wave. In his method, a triangular shape of the flow wave was assumed to determine the timing features of arterial pressure. Hence, the reflection index (RI) derived by Westerhof's method can be calculated via BP only [6].

 On the other hand, pulse wave velocity (PWV) is another popular method for the quantification of aortic stiffness [7]. The most widely used method for determining PWV is to measure the time delay between characteristic points on two pressure waveforms that are a known distance apart. Recently, an innovative analysis algorithm of multimodal pressure flow (MMPF) was proposed to trace the interaction between BP and blood flow using the phase shift of spontaneous oscillations [8–10]. In this study, it is assumed that the ECG can present the activating potential of heart beating and it is measured as the driving signal for the cardiovascular system [11]. In addition, BP performs as the output signal of the cardiac cycle, which reflects complicated responses of the overall cardiovascular system. Thus, a new application of multimodal analysis was proposed to investigate the interactive phase shift between ECG and BP during a cardiac cycle. The assumption made in this study is that the phase shift between intrinsic components of cardiac oscillations extracted from recordings of ECG and BP reflects the systemic resistance of a cardiovascular system. Therefore, signal processing techniques for decomposing the intrinsic components from ECG and BP signals are critical for these new applications.

Methodologically, there are many different signal processing methods that perform high-efficiency signal decomposition, such as independent component analysis (ICA) [12] and wavelet decomposition [13]. ICA contributes to the applications of blind signal separations based on statistical characteristics of the signals, which reflect linear combinations of different signal sources. Wavelet decomposition offers simultaneous interpretation of the signal in both time and frequency that allows local, transient, intermittent components to be calculated. However, such traditional signal processing method is based on linear assumption. The components derived by wavelet decomposition are often obscured due to the inherent averaging. In 1998, Huang et al. proposed the innovative algorithm of EMD signal decomposition, in which the components are decomposed adaptively to the nature of signals but not the base of transformation [14]. Theoretically, each intrinsic mode function (IMF) decomposed by EMD reflects the response actuated by the corresponding activity of a particular underlying physiological mechanism. In practices, the unpredictable intermittent turbulences damage the consistencies of IMFs. This phenomenon is noted as mode mixing. Recently, an ensemble empirical mode decomposition (EEMD) has been introduced which is considered as an enhanced algorithm of EMD, which solves the problem of mode mixing in the original EMD [1]. In this pilot study, it is assumed that the reflected waves of BP can be derived as a particular intrinsic component (i.e., IMF) by EEMD. Hence, a new EEMD-based calculation of RI can be achieved. Moreover, EEMD also works to decompose the cardiac oscillations from ECG and BP in the new application of multimodal analysis. Phase shift between the cardiac oscillations of ECG and BP is considered to be a phase delay between the driving signal (i.e., ECG) and the output signal (i.e., BP) of the cardiovascular system. It is considered as a new assessment of systemic impedance of the cardiovascular system which is the second EEMD-based assessment presented in this study.

Finally, Pearson's correlation coefficient was applied to check the correlations between SAP, EEMD-based RI, and the phase shift (between ECG and BP on cardiac oscillation). According to the results of the correlation analysis, EEMD-based RI acts as an indicator of arterial stiffness, showing significant positive correlation with SAP and significant negative correlation with the phase shift between ECG and BP on cardiac oscillation. The phase shift between ECG and BP on cardiac oscillation also acts as another indicator for systemic resistance of a cardiovascular system, which has a negative correlation with SAP. These two indicators show two different profiles of the cardiovascular system and have significant negative correlations with each other. Moreover, correlations between SAP (a direct measurement of BP), RI (a secondary parameters depends on the waveform of BP), and phase shift between ECG and BP (a phase delay between two different signals) show different profiles of the cardiovascular system and significant connections among them.

## 2. Material

In this investigation, the study material (i.e., ECG and BP recordings) was recorded during an animal experiment, which was approved by the Animal Research Ethics Review Committee of the Far Eastern Memorial Hospital in Taiwan. In this experiment, a male Lanyu-50 pig with body weight of around 10–15 kg was the subject. After intramuscular injection of Zoletil (Zoletil 50 Vet; Virbac S.A., Carros, France) 3–5 mg/kg, an intravenous line was established in the vein behind the ear. An oximeter was applied on the tail. Other monitored biosignals included body temperature and ECG. Body temperature was maintained by a heating blanket and warm air. Additional Zoletil was prepared to achieve immobility before intubation. After intubation and confirming the position of the endotracheal tube (size 5.0–5.5 mm internal diameter), 4 mg pancuronium was injected intravenously. Subsequently, 5 mg/kg Zoletil and 4 mg pancuronium were given hourly. The pig was anesthetized following the same procedures above, with additional central venous catheter (20G-22G-22G, BD) at the right internal jugular vein and an arterial catheter (20G) at the left femoral artery under cut-down procedure. Lactate Ringer's solution, Hespander, and whole blood (donated from other pigs) were administered to maintain adequate volume status (central venous pressure >5 mmHg) and hemoglobin level (>8 g/dL). Norepinephrine or epinephrine (bolus or continuous infusion) can be administered as required to maintain systolic blood pressure >100 mmHg, especially after graft reperfusion. At the end of the surgery, if the hemodynamic profile was stable, weaning from ventilator support can be attempted.

To generate the ECG and BP recordings during clamping-relaxing-clamping-relaxing of the intestinal artery, the pig's intestinal artery was blocked by clamping briefly (e.g., one minute) and then relaxing the clamping to produce successive time series recording under different situations and transition state between them. This designed process was run twice consecutively to derive four-minute recordings of ECG and BP. The raw data of ECG and BP were measured by IntelliVue MP60 (Philips), an multichannel physiological monitoring system usually equipped in surgical operation rooms and intensive care units. The data was measured and stored at sampling rate of 1000 Hz and length of 240,000 sample points. No preprocessing algorithms had been applied to the raw data recorded by the MP 60 before further analysis.

## 3. Methods

### 3.1. Empirical Mode Decomposition (EMD)

Empirical mode decomposition (EMD) performs an adaptive method to remove oscillation successively though repeatedly subtraction of the envelope means [14]. To a signal *x*(*t*), the EMD algorithm consists of the following steps.

1. Connect the sequential local maxima (respective minima) to derive the upper (respective lower) envelop using cubic spline.
2. Derive the mean of envelope, *m*(*t*), by averaging the upper and lower envelopes.
3. Extract the temporary local oscillation *h*(*t*) = *x*(*t*) − *m*(*t*).
4. Repeat the steps of 1–3 (i.e., the sifting process) on the temporary local oscillation *h*(*t*) until *m*(*t*) is close to zero. Then, *h*(*t*) is an IMF noted as *c*(*t*).
5. Compute the residue *r*(*t*) = *x*(*t*) − *c*(*t*).
6. Repeat the steps from (1) to (5) using *r*(*t*) for *x*(*t*) to generate the next IMF and residue.

Therefore, the original signal *x*(*t*) can be reconstructed using the following formula:

(1) x(t)=∑i=1nci(t)+rn(t),

where *c*_*i*_(*t*) is the *i*th IMF (i.e., local oscillation) and *r*_*n*_(*t*) is the *n*th residue (i.e., local trend).

As the algorithm uses all the local extremes to construct the envelopes, the mode mixing would be inevitable when the signal contains intermittent processes. As discussed by Wu and Huang [1], the intermittence would cause the resulting true physical processes to be obscured by the fragmentation of a given signal.

### 3.2. Ensemble Empirical Mode Decomposition (EEMD)

**Figure 1:**
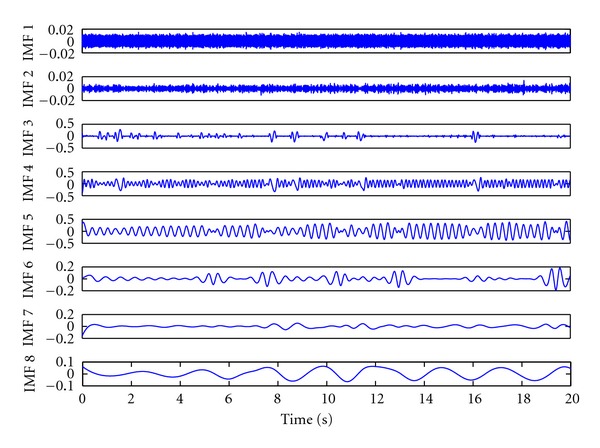
First 8 IMFs derived from a 12-second recording of a pig's BP by the original technique of EMD. Significant mode shifting can be observed in IMFs 4-5, which reflect inconsistencies in mode functions.

**Figure 2:**
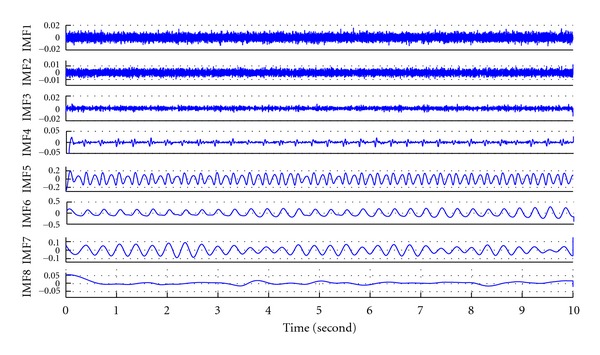
First 8 IMFs derived from a 12-second recording of a pig's BP by the noise-assisted technique of EEMD.

EMD is an iterative signal processing algorithm which decomposes the IMFs from the signal by the iterative sifting processes [14]. The essential algorithm of EMD is associated with a major difficulty of mode mixing.  Figure 1 shows first 8 IMFs decomposed from a pig's BP recording by the original technique of EMD. Significant phenomenon of mode mixing can be observed in IMF 4–6, which perform inconsistencies in mode functions. Recently, Wu and Huang proposed EEMD as a noise-assisted data analysis method to overcome mode mixing problem [1]. In EEMD, white noise is added into the original signal to generate the mixtures for decompositions by EMD. Ensemble IMFs can be derived by averaging the IMFs decomposed from the mixtures. Since the intermittent fluctuations, which cause mode mixing problem, are coupled with the added white noise to be filtered, the problem of mode mixing has been effectively solved in EEMD.  Figure 2 shows first 8 IMFs decomposed from the same recording by the noise-assisted technique of EEMD. The problem of mode mixing was solved and IMFs present consistencies in mode functions.

### 3.3. Monte Carlo Verification and Noise Removal

Monte Carlo simulation is a computational algorithm that relies on repeated random sampling to compute their results. In the confidential test of EMD, the repeated numerical simulations to characterize the properties of random noises applied to EMD can be based on the application of Monte Carlo simulation. Then, the confidential zone of IMFs decomposed from random noises can be defined by Monte Carlo simulations. An IMF with properties out of the confidential zone can be verified as a dominant component of the signal. This approach for verifying the dominant components of the signals is noted as Monte Carlo verification [2, 15]. Monte Carlo verification works to verify the IMFs contributed by noise or the dominant signal. The high-frequency noise of real-world signals can be reconstructed via the noisy components verified by the Monte Carlo verification, and the main waveform of signals can be reconstructed by the rest of intrinsic components and residual.

 In the Monte Carlo verification, two parameters of energy density and averaged period for each IMF should be calculated using the following equations [16]:

(2) En=1N∑j=1N[Cn(j)]2,T¯n=∫Sln⁡T,ndln⁡T(∫Sln⁡T,ndln⁡TT)−1,

where *C*_*n*_(*j*) is the *j*th sample of the *n*th IMF, *E*_*n*_ is the energy density of the *n*th IMF, *S*_ln⁡*T*,*n*_ is the Fourier spectrum of the *n*th IMF as a function of ln⁡*T*, *T* is the period, and ${\bar{T}}_{n}$ is the averaged period of the *n*th IMF.

**Figure 3:**
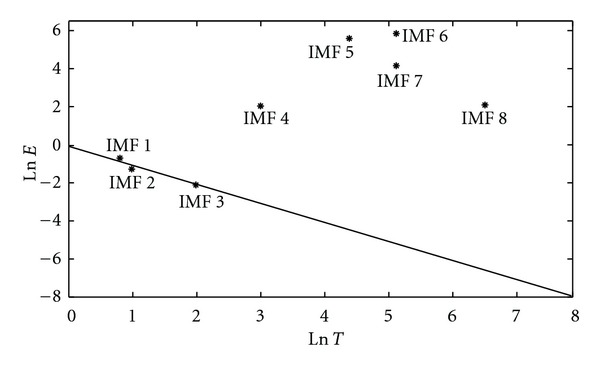
Logarithmic energy density-averaged period plot for the first 8 IMFs decomposed from pig's blood pressure signal.

**Figure 4:**
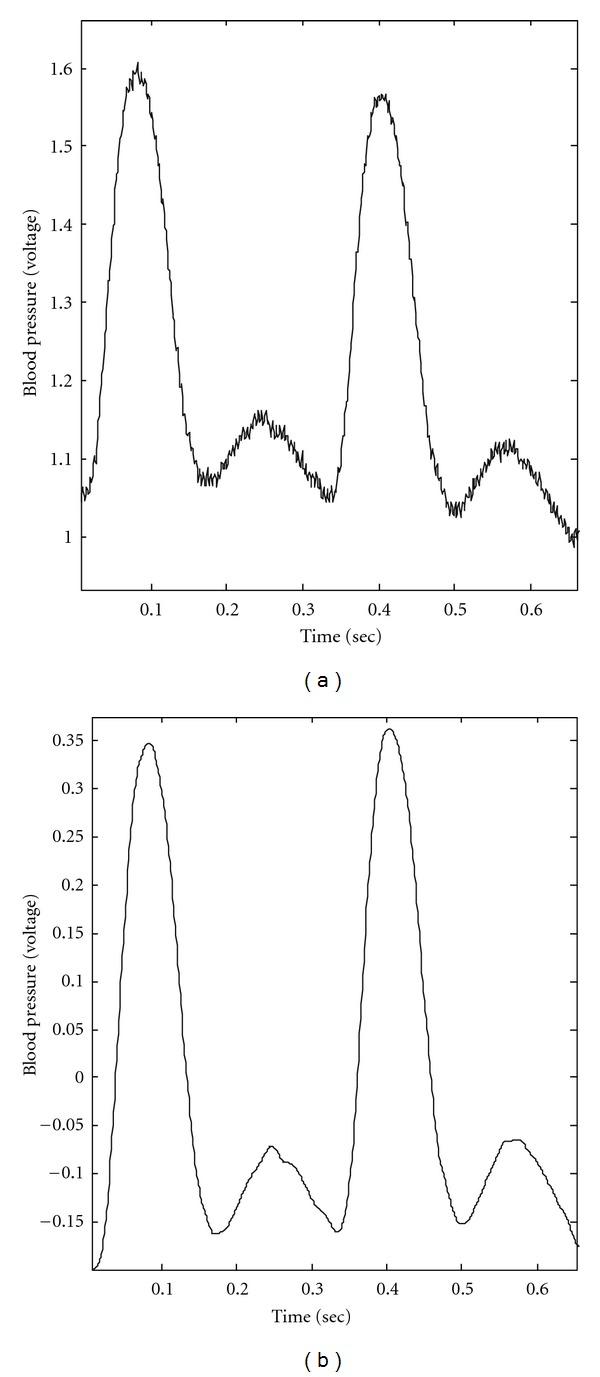
The original pulses and the reconstructed pulses of BP. (a) The original pulses of a pig's BP. (b) The reconstructed pulses of a pig's BP, which are reconstructed via the IMFs 4–7.

On the logarithmic energy density/averaged period plot as shown in Figure 3, the first 3 IMFs can be fitted by a straight line with negative slope. According to the characteristics of white noise and fractal Gaussian noise derived by EMD [16–18], logarithmic energy density/averaged period plot for IMFs decomposed from a Gaussian noise is similar to a straight line with negative slope. Thus, the high-frequency noisy components are considered as the first *n* IMFs, which have a distribution of logarithmic energy densities and averaged periods similar to a straight line with negative slope value in the Monte Carlo verification. In Figure 3, the first 3 IMFs are verified as the noisy components of blood pressure signals. Moreover, IMF 8 has an averaged frequency of 0.46 Hz, which is induced by the activity of an unidentified physiological mechanism with lower frequency band than that of the basic cardiac cycle. Hence, the main waveform of blood pressure signal can be constructed via IMFs 4–7. In Figure 4, the reconstructed pulses of BP have main waveforms similar to the original pulses but excluding high-frequency noise and baseline shifting.

### 3.4. The EEMD-Based Calculation for RI

Augmentation index (AIx) is an assessment of wave reflection and an indicator of aortic stiffness [4, 5]. Unfortunately, the inflection points on systolic peaks are not distinguishable, and so AIx cannot be obtained easily in this study. Recently, Westerhof demonstrated a new quantification method for wave reflection in the human aorta [6]. They assumed a triangular wave to simulate the extra measurement of aortic blood flow, with duration equal to ejection time, and to get approximations of the inflection points of BP using the time point of 30% of ejection time. In this study, the calculation of RI using the assumption of 30% ejection time is noted as the referred calculation of RI. Magnitudes of forward wave (*P*_*f*_) and reflected backward wave (*P*_*b*_) are separated using the magnitude of BP at the inflection point and the secondary rising magnitude of BP. Then, the reflection index (RI) is defined as

(3) RI=PbPb+Pf.

**Figure 5:**
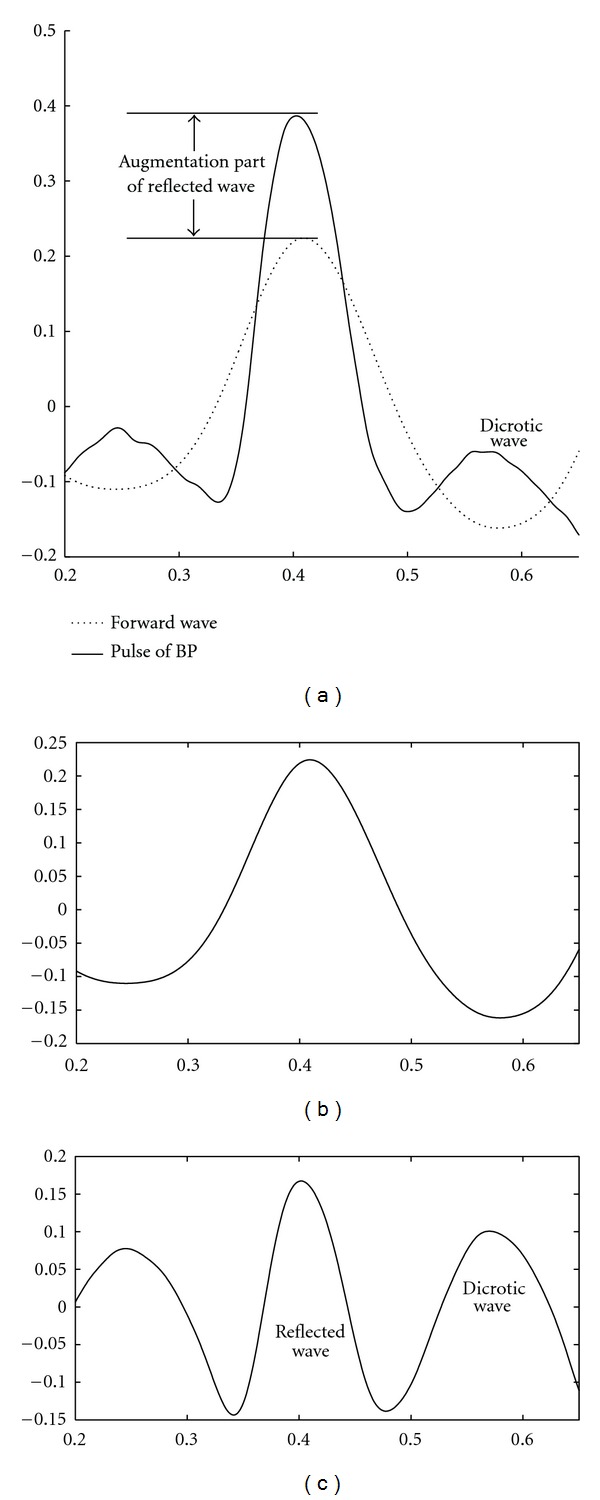
Illustration of a reconstructed pulse of a pig's BP. A whole pulse is assumed to be the ensemble of the forward wave and two riding waves (i.e., reflected wave and dicrotic wave). (a) Solid line shows the reconstructed forward wave of a pig's BP and the dash line shows complete waveform. (b) Assumed forward wave, which was reconstructed via IMFs 4, 6, and 7; (c) IMF 5 contains the reflected wave and dicrotic wave.

In the EEMD-based calculation of RI, IMFs 1–3 decomposed from BP had been verified as high-frequency noisy components using the Monte Carlo verification [15]. Thus, complete pulses of the pig's BP can be reconstructed via IMFs 4–7. IMF 4 contributes a high-frequency part of BP with small amplitude. Sometimes, an intrinsic component of the original signal coupling with different added white noises can be decomposed into two different IMFs in EEMD. Then, two IMFs present a very high value of Pearson's correlation coefficient and can be merged together as single IMF. In this investigation, Pearson's correlation coefficient between IMFs 6 and 7 is 0.825 and the averaged frequencies are similar. Therefore, these 2 IMFs can be combined as an intrinsic component. Moreover, IMF 5 presents double in the number of peaks compared to the number of heart beats, as the same number of fluctuating cycles of the combination of IMFs 6 and 7. Half of the peaks of IMF 5 accompany the systolic peak of BP, and the other half accompany the dicrotic peaks of BP. Theoretically, the decomposition of EEMD is adaptive to the waveform of the signal; the separation between IMF 5 and its corresponding residue is sensitive to the discontinuous point on the systolic peak of BP as the inflection point. Therefore, the reconstructed wave via IMFs 4, 6, and 7 presents the basic fluctuation pattern of BP. And IMF 5 contributes the appended part of BP as the combination of reflection wave and dicrotic wave. In this investigation, the reconstructed wave via IMFs 4, 6, and 7 is considered as the forward wave as shown in Figure 5(a). It is also assumed that IMF 5 contributes the reflected wave and the dicrotic wave as two riding waves on the forward wave of BP.  Figure 5(b) illustrates the forward wave only, and Figure 5(c) illustrates IMF 5, which contains the reflected wave and dicrotic wave. The forward wave follows the same rhythm as the heartbeat and presents the main cardiac oscillation of BP. IMF 5 contains the reflected wave and the dicrotic wave and shows an averaged frequency of oscillation twice that of the cardiac oscillation. Thus, the magnitude of the reflected wave (*P*_*b*_) was defined as the amplitude of the reflected wave in IMF 5. In addition, the magnitude of forward wave (*P*_*f*_) was measured using the amplitude of the reconstructed forward wave.

### 3.5. Phase Shift between ECG and BP on Cardiac Oscillation

Cerebral autoregulation controls dilatation and contributes to the constriction of the arterioles to maintain blood flow in response to changes of systemic blood pressure [19]. Therefore, a multimodal analysis algorithm was used to assess autoregulation mechanism by quantifying nonlinear phase interactions between spontaneous oscillation in blood pressure and flow velocity [8, 9]. Multimodal analysis acts to trace the phase delay between the spontaneous oscillations extracted from two different physiological signals (i.e., blood pressure and blood flow in the pioneering application).

**Figure 6:**
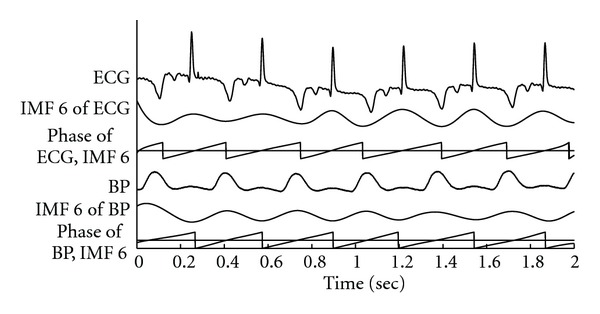
Illustration of phase shift between cardiac oscillations extracted from ECG and blood pressure. Plots from top to bottom are ECG signal, IMF 6 derived from ECG, time-phase distribution of ECG cardiac oscillation, BP, IMF 6 derived from BP, time-phase distribution of BP cardiac oscillation. Phase shift can be observed as the difference between the accumulative time-phase distributions of cardiac oscillations for ECG and BP.

In this investigation, ECG and BP are treated as the driving and output signals of the cardiovascular system. As a system defined in the field of digital signal processing, system impedance causes the decay ratio and phase delay between the output and the input. Phase shift between ECG and BP reflects the phase delay between the input and output of a human cardiovascular system. Peaks of IMF 6 decomposed from ECG present the R points of ECG signal, and peaks of IMF 6 decomposed from BP present the peaks of systolic wave. Therefore, phase shift between ECG and BP also presents a ratio between the pulse transit time (i.e., transit time between R peaks of ECG and peaks of systolic blood pressure) and heartbeat interval. It is assumed that the interactive phase shift (phase delay) between ECG and BP on the cardiac oscillation reflects the phase delay caused by the systemic impedance of the cardiovascular system. To determine the intrinsic components (i.e., IMFs) which reflect the cardiac oscillations of BP and ECG, the pig's ECG and BP recordings are decomposed into the first 9 IMFs.  Table 1 shows the averaged frequencies of IMFs 5–9 for ECG and BP. Average frequency of IMF contributes as a clue to find the corresponding physiological mechanism for each component. In contrast to the human heartbeat rhythm, a young pig's heartbeat is much quicker than that of a human. Average frequency of a pig's heartbeat is around 3 Hz. Therefore, the cardiac oscillations were identified as the 6th IMFs for both ECG and BP. Furthermore, Hilbert transform was used to derive the time-amplitude-phase distribution from the cardiac oscillations [8–10].  Figure 6 illustrates the evaluated phase shift between ECG and BP on cardiac oscillation. The cardiac oscillation of ECG was defined as the IMF with rhythm similar to heart beating, as IMF 6 derived from ECG. And the cardiac oscillation of BP was defined as the IMF with rhythm similar to the occurrence rhythm of systolic peak, as IMF 6 derived from BP. Then, the accumulative time-phase distributions can be via the time-phase distributions shown in Figure 6. Therefore, the phase shift is defined as the difference between the accumulative phases for every time point.

### 3.6. Pearson's Correlation Coefficient

Pearson's product-moment correlation coefficient is a measurement to identify the linear relationship between two variables [20]. In Pearson's correlation coefficient, the value of 1 indicates a perfect linear relationship between two variables and a negative correlation is indicated by the value of −1.

The traditional interpretation of a correlation coefficient uses five “rules of thumb” to interpret the correlation between two variables as follows [21]:

 0.20 > |*r*| > 0 as *negligible correlation, *
 0.40 > |*r*| > 0.20 as *low correlation, *
 0.60 > |*r*| > 0.40 as *moderate correlation, *
 0.80 > |*r*| > 0.60 as *significant correlation, *
 1.00 > |*r*| > 0.80 as *high correlation. *

A positive value of correlation coefficient represents a positive correlation between two variables and a negative one presents a negative correlation. In this study, the value of correlation coefficient is interpreted using such interpretation rules.

## 4. Results

**Figure 7:**
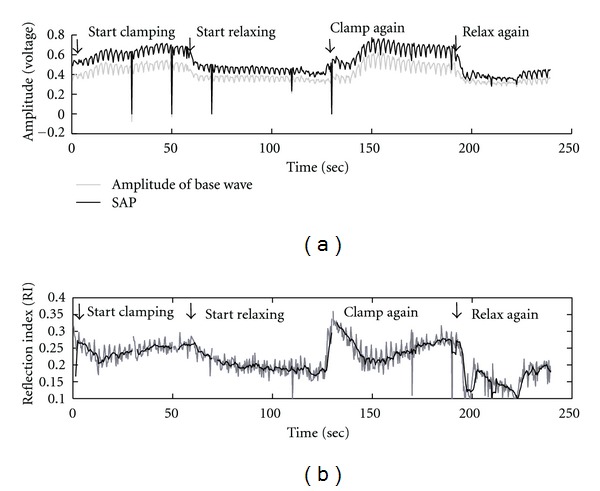
The analysis results of EEMD-based RI. (a) SAP and the magnitude of forward wave. (b) The original and filtered EEMD-based RI.

**Figure 8:**
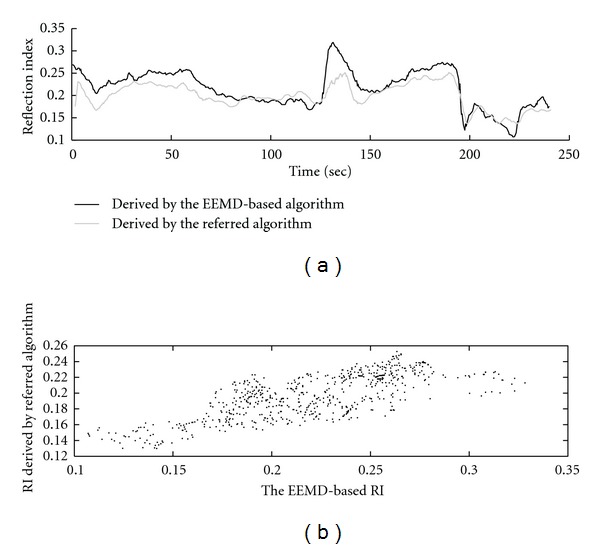
Comparisons between the analysis results of RI using different algorithms. (a) The time-sequence plot of RI during the simulated surgical operation. (b) The distribution of the referred RI against the EEMD-based RI.

The analysis results of EEMD-based RI and progression of SAP as well as the magnitude of the forward wave of BP during the simulated surgical operation are shown in Figure 7. According to the results, it is shown that SAP rises and then remains steady on a high level during the period of artery clamping then falling during the period of arterial relaxing as shown in Figure 7(a). Moreover, it is also shown that there are cyclic changes in SAP and in the magnitude of forward wave. To verify the underlying physiological mechanism causing the cyclic changes, the number of cycles were counted and found that the average period of the cyclic change of SAP is 2.92 seconds (with average frequency of 0.34 Hz), which performs a rhythm similar to the respiration rate according to our observation. Moreover, the cyclic changes in SAP and in the magnitude of the forward wave also affect the values of RI, which also contains cyclic changes in values. To eliminate the effect caused by the interaction between respiration and the heartbeat, EEMD-based RI was filtered using a moving average filter (9 samples have been used for the moving average filter, since the average number of heartbeats during a cyclic change of SAP is around 9).  Figure 7(b) shows the original and filtered EEMD-based RI. Furthermore, the same calculations of RI were repeated using the referred algorithm proposed by Westerhof et al., and compared to the EEMD-based results. In Figure 8(a) the two different RI are presented by time-sequence plots. Furthermore, the distribution of the two different RIs is shown in Figure 8(b). A positive correlation has been observed between the two RIs (*r* = 0.759).

**Figure 9:**
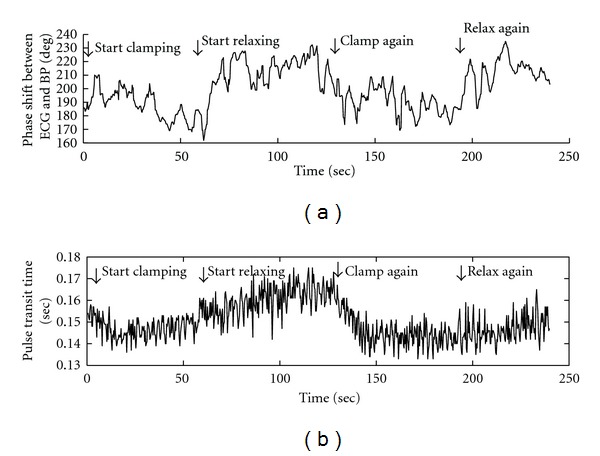
Phase shift between ECG and BP on cardiac oscillation.

In addition, multimodal analysis was conducted to investigate the systemic resistance in the cardiovascular system using the phase shift between ECG and BP on the cardiac oscillation. Due to the sensitivity of the Hilbert spectrum, the phase shift between two cardiac oscillations is not constant. Therefore, phase shift was also filtered by a moving average filter (the number of points used for moving average filter is 100, which is the equivalent cut-off frequency of 10 Hz for the sampling rate of 1000 Hz). The phase shift between ECG and BP on cardiac oscillation is shown in Figure 9(a). For the purpose of comparison with the analysis results of phase shift between ECG and BP, pulse transit time (PTT) between ECG and BP was analyzed.  Figure 9(b) shows the analysis result using PTT. Phase shift is found to be more sensitive to the changes of manual control conditions (i.e., actions of clamping and relaxing). Analysis results of PTT are shown in Figure 9(b). PTT reflects the time delay between the R peak of ECG and the systolic peak of BP. The phase shift presented by the phase delay is different from the time delay presented by PTT. According to the plots shown in Figure 9, the phase shift is found to be more sensitive to the manual control actions than that presented by PTT.

**Figure 10:**
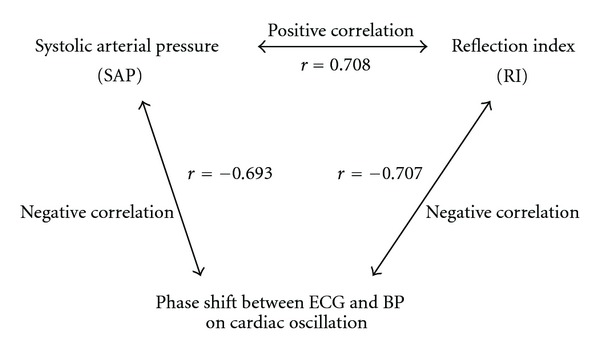
Illustration of the correlations among SAP, RI, and phase shift between ECG and BP on cardiac oscillation.

For further comparisons among the original physiological signals (i.e., SAP) and the physiological indexes (i.e., phase shift between ECG and BP on cardiac oscillation and two different RIs derived by the EEMD-based and the referred algorithms), correlation coefficients were used to evaluate the correlations between the two different physiological signal/index.  Table 2 shows the values of correlation coefficients for correlations of one-to-one comparisons. According to the results shown in Table 2, the two different assessments of RI have a positive correlation since they similarly perform as indicators for arterial stiffness. RI has a positive correlation with SAP, and phase shift between ECG and BP has a negative correlation with SAP. Furthermore, Figure 10 shows interesting correlations among SAP, RI, and phase shift between ECG and BP on cardiac oscillation.

## 5. Discussions and Conclusions

In previous studies, there were many different physiological parameters (such as pulse transit time, augmentation index, and reflection index) developed to investigate humans' cardiovascular systems using traditional algorithms based on linear assumption. However, since the human cardiovascular system is nonlinear and nonstationary, 4 necessary conditions (i.e., complete, orthogonal, local, and adaptive) should be considered in system analysis. Recently, EEMD proposed as an innovative analysis algorithm, which had been developed to satisfy the 4 conditions, is considered as a better solution to develop new assessments for cardiovascular system. Therefore, this approach has been considered to develop EEMD-based algorithms for cardiovascular system evaluation. This study did not provide satisfiable number of cases to prove any clinical findings. However, the EEMD-based analysis algorithm is computing extensive and time consuming. Hundred times of EMD are required in an EEMD decomposition to diminish the residue of added white noises. Therefore, EEMD-based analysis algorithms are hard to implement in an embedded system and applied to online monitoring system.

In the practical applications of EMD and EEMD, which algorithm fits the requirements of decomposition to nonlinear and nonstationary signals is still a critical issue. IMFs decomposed by the original EMD can conserve the characteristics of nonlinearity well in mode functions. However, mode mixing is a weakness of EMD in applications for extracting any mode functions with particular physical or physiological meanings. In contrast EEMD works to solve the problem of mode mixing. But characteristics of nonlinearity for mode functions can be destroyed in the ensemble form of IMFs. In this study, extracting intrinsic components with consistent characteristics in modulation is more important than conserving the characteristics of nonlinearity in the mode functions. Therefore, EEMD was applied in this investigation. What kind of characteristics should be conserved in the IMFs determines the use of EMD or EEMD.

In this study, an animal experiment was conducted for simulating changes in the cardiovascular system using a designed process to generate study material. In this one-animal experiment, relationships among different parameters are considered purely and directly. Influences caused by individual can be ignored in this investigation. Moreover, SAP is considered as a directly physiological measurement, EEMD-based RI is a secondarily derived parameter from BP, and phase shift between ECG and BP is a correlated phase delay between two physiological measurements. Therefore, connections among SAP, EEMD-based RI, and phase shift between ECG and BP are considered to reflect interactions of different physiological mechanisms in the human cardiovascular system.

According to the results, EEMD-based RI and phase shift between ECG and BP are significantly correlated with SAP. Furthermore, the correlation between these two parameters is also significant. It contributes an evidence for interactions among SAP, arterial stiffness, and systemic resistance of cardiovascular system. Moreover, this pilot study aims to present the functions of these two presented analysis techniques based on EEMD but not the physiological findings. Hence, in order to make a contribution for understandings of underlying mechanisms of humans' cardiovascular systems, further study should be conducted with a sufficient number of animal experiments in the future works. Furthermore, mutual information analysis provides a powerful tool to verify the dependence between the two variables [22]. For the purpose of detailing the connections and dependencies among those parameters, the mutual information criteria should be considered and applied in future work.

Moreover, both the referred and the EEMD-based algorithms of RI evaluation present an interesting phenomenon during the period of artery clamping as shown in Figure 8. The value of RI eruptively increases at the instant of artery clamping and falls down at the first 20 seconds during artery clamping. Then, the RI arises again and becomes steady. During the periods of artery relaxing, the changes of RI values present much smoother patterns than those during the clamping periods.

In addition, IMFs decomposed by EEMD are ensembles of many EMD decompositions to mixtures of the signal and different added white noises. A complicated signal, which contains many intrinsic mode functions, coupled with different added white noise to generate different combinations of IMFs in EEMD. Therefore, an intrinsic component of the original signal may appear with different orders in different EMD decompositions because of coupling with different added noises. Two IMFs sharing the same frequency can be resulted when an intrinsic component is decomposed into two IMFs evenly in EEMD. In Figure 2, IMFs 6 and 7 sharing the same frequency are a good example for this phenomenon. This is not a difficult problem to deal with. An orthogonal test to two successive IMFs is helpful to verify this phenomenon. The two IMFs sharing the same frequency can be merged together as a single IMF.

Finally, the referred algorithm of RI analysis is based on the triangular method to separate reflective and forward waves of BP. This method was derived and validated in central aorta but not femoral aorta. In this investigation, EEMD was considered to perform an adaptive algorithm in intrinsic component separation. Reflective and forward waves of BP are considered as two intrinsic components of BP with slight phase delay and difference in waveforms. EEMD works to separate these two components adaptively to the waveform of BP. The referred algorithm is considered to be as a criterion of inflection point determination without validation for BP signals derived from femoral aorta. The analysis results by the referred algorithm were used to be compared with the analysis results by EEMD-based method. In practical applications, the referred algorithm based on triangular method in femoral BP analysis should be validated.

## Figures and Tables

**Table 1:** Averaged frequencies of IMFs 5–9 decomposed from a pig's ECG and blood pressure by EEMD.

	ECG	Blood pressure
IMF 5	7.19 Hz	6.20 Hz
IMF 6	3.10 Hz	3.09 Hz
IMF 7	2.16 Hz	2.98 Hz
IMF 8	1.12 Hz	0.46 Hz
IMF 9	0.47 Hz	0.24 Hz

**Table 2:** Correlations among the SAP, RI, and phase shift. According to the interpretation rules used in this study, 0.6 > *r* > 0.4 represents a moderate correlation and 0.8 > *r* > 0.6 represents a significant correlation.

Physiological signal/index		Correlation coefficient	Correlation
EEMD-based RI	Referred RI	0.759	Positive and significant
EEMD-based RI	Phase shift	−0.707	Negative and significant
Referred RI	Phase shift	−0.543	Negative and moderate
SAP	EEMD-based RI	0.708	Positive and significant
SAP	Referred RI	0.731	Positive and significant
SAP	Phase shift	−0.693	Negative and significant
